# Atomic-Scale Insights into Hydrogen-Induced Ductile-to-Brittle Transition in Polycrystalline α-Fe with Varying Hydrogen Concentrations and Grain Sizes

**DOI:** 10.3390/ma19194157

**Published:** 2026-09-29

**Authors:** Peifen Yao, Yaoyinqi Wang, Qiaosheng Zhang, Shengde Di, Xiaoming Luo

**Affiliations:** 1 College of Pipeline Engineering, Xi’an Shiyou University, Xi’an 710065, China; 2 Research Institute of Petroleum Engineering Technology, SINOPEC Shanghai Offshore Oil & Gas Company, Shanghai 200120, China; wyyqye1@163.com; 3 Changqing Engineering Design Co., Ltd., China National Petroleum Corporation, Xi’an 710018, China; qiaoszhang@163.com; 4 College of Pipeline and Civil Engineering, China University of Petroleum (East China), Qingdao 266580, China; z24060056@s.upc.edu.cn

**Keywords:** hydrogen embrittlement, dislocation, critical concentration, ductile-to-brittle transition, molecular dynamics

## Abstract

**Highlights:**

- MD simulations revealed hydrogen-induced fracture transition in polycrystalline α-Fe.
- Grain-boundary H accumulation inhibited dislocation emission and promoted DBT.
- DBT occurred at a critical grain-boundary hydrogen concentration of 7–9%.
- A method was developed to estimate critical hydrogen concentration from grain size.

**Abstract:**

With the development of hydrogen pipelines, hydrogen embrittlement has become a critical issue affecting the safe service of pipeline steels. However, the critical hydrogen concentration for the hydrogen-induced ductile-to-brittle transition and its dependence on grain size remain unclear. Molecular dynamics simulations were performed to elucidate the effects of hydrogen concentration and grain size on the hydrogen-induced ductile-to-brittle transition by investigating hydrogen diffusion, dislocation emission, and fracture evolution in polycrystalline α-Fe. The results demonstrate that, with increasing hydrogen concentration, hydrogen atoms preferentially diffuse along grain boundaries and gradually reach segregation saturation, resulting in continuous degradation of mechanical properties. The initial fracture strain of both fine-grained and coarse-grained models decreases by more than 40%, while fracture energy decreases by more than 50%. Crack surface areas increase by factors of 2.48 and 1.86, respectively, indicating greater hydrogen sensitivity of the fine-grained model. At grain-boundary hydrogen concentrations of approximately 7–9%, suppressed grain-boundary dislocation emission triggers the transition from plastic deformation to brittle intergranular fracture, marking the onset of the hydrogen-induced ductile-to-brittle transition. A quantitative relationship between grain size and grain-boundary atom fraction was established to predict the critical hydrogen concentration for this transition. This study reveals the role of hydrogen concentration in regulating dislocation emission and intergranular fracture, providing insights for hydrogen embrittlement assessment and pipeline material design.

## 1. Introduction

With the global transition toward low-carbon energy systems and the increasing implementation of carbon neutrality strategies, hydrogen energy has been widely regarded as a critical component of future energy infrastructures due to its potential for zero-carbon utilization, high energy density, and compatibility with renewable energy integration [1,2,3]. However, the large-scale adoption of hydrogen energy is still restricted by challenges associated with efficient storage and transportation, among which the development of long-distance, high-capacity, and economically viable hydrogen delivery technologies remains a major barrier for industrial-scale deployment. Compared with high-pressure gaseous hydrogen and liquid hydrogen transportation, pipeline-based hydrogen transportation provides distinct advantages, including large transmission capacity, continuous operation, low transportation costs, and compatibility with the repurposing of existing natural gas pipeline networks. Consequently, hydrogen pipelines are considered one of the most promising solutions for future large-scale hydrogen transmission [4,5]. Nevertheless, the introduction of hydrogen into pipeline systems raises critical concerns regarding the long-term integrity of metallic pipeline materials. Prolonged exposure to high-pressure hydrogen environments can induce hydrogen embrittlement, resulting in significant degradation of mechanical properties, such as reduced ductility, impaired fracture toughness, and compromised structural reliability. Therefore, elucidating the hydrogen embrittlement mechanisms of pipeline steels, revealing the intrinsic correlation between hydrogen accumulation and fracture behavior, and determining the critical hydrogen concentration associated with safe operation are of essential scientific and engineering importance for ensuring the long-term safety and reliability of hydrogen transportation infrastructure.

Under hydrogen exposure, hydrogen molecules undergo adsorption and dissociation on the metal surface, followed by the absorption and diffusion of atomic hydrogen into the metallic lattice. Once introduced into the material, hydrogen atoms interact with lattice defects, stress fields, and microstructural heterogeneities, leading to preferential diffusion and accumulation at regions with high hydrogen trapping capacity, including grain boundaries, dislocations, vacancies, and crack tips [6]. The localized enrichment of hydrogen in these regions can significantly modify plastic deformation behavior and fracture evolution, ultimately resulting in hydrogen embrittlement. To date, several mechanisms have been proposed to describe hydrogen embrittlement, among which hydrogen-enhanced localized plasticity (HELP), hydrogen-enhanced decohesion (HEDE), and the hydrogen bubble theory (HBT) are widely recognized as representative models [7,8]. Nevertheless, owing to the complex coupling between hydrogen transport, microstructural characteristics, and local stress states, these mechanisms are not mutually exclusive but may coexist and compete, with their relative contributions varying according to material composition, hydrogen concentration, and mechanical loading conditions [9,10]. Therefore, revealing the hydrogen concentration-dependent evolution of microscopic deformation mechanisms and establishing the transition criteria among different embrittlement mechanisms remain critical challenges for advancing the fundamental understanding and safe application of hydrogen-related infrastructure.

The HELP theory proposed by Beachem [11] suggests that hydrogen atoms accumulated in stressed crack-tip regions can reduce the energy barrier for dislocation motion, thereby promoting dislocation emission, localized plastic deformation, and subsequent crack propagation. This mechanism has been widely recognized as one of the fundamental models explaining hydrogen-induced plasticity enhancement [12,13]. Extensive experimental studies and atomic-scale simulations have further demonstrated that hydrogen can strongly regulate dislocation nucleation, mobility, and interactions with grain boundaries. Nevertheless, recent evidence has challenged the conventional assumption that hydrogen universally enhances dislocation activity, indicating that its influence is highly dependent on local hydrogen concentration, stress state, and microstructural characteristics. At relatively low hydrogen concentrations, hydrogen has been reported to facilitate dislocation nucleation and migration in α-Fe, increase local dislocation density, and promote grain-boundary activation through enhanced dislocation–grain-boundary interactions [14,15]. In contrast, under highly hydrogen-enriched conditions, hydrogen may suppress dislocation motion through solute drag effects or dislocation pinning mechanisms [16,17,18,19]. For instance, Song and Curtin [17] demonstrated that hydrogen atmospheres surrounding mobile dislocations can reduce dislocation mobility and weaken crack-tip blunting capability. Matsubara [18] further revealed that hydrogen-induced inhibition of grain-boundary dislocation emission plays a critical role in intergranular crack initiation, while Zhang et al. [19] confirmed that hydrogen-induced dislocation pinning promotes brittle fracture in nanocrystalline materials. These findings suggest that the role of hydrogen in regulating dislocation behavior may undergo a concentration-dependent transition from promoting plastic deformation to restricting dislocation activity. However, whether this transition is governed by a quantitative critical hydrogen concentration and how this threshold is affected by grain structure remain unresolved, requiring systematic investigation.

In addition to hydrogen–dislocation interactions, the coupling between hydrogen redistribution and stress fields represents another fundamental factor governing hydrogen embrittlement evolution. Previous studies have demonstrated that hydrogen diffusion is strongly affected by hydrostatic stress gradients, causing hydrogen atoms to migrate toward and accumulate in regions with high hydrostatic tensile stresses, thereby establishing a coupled relationship between local hydrogen enrichment and stress concentration fields [20,21]. Such hydrogen–stress interactions can further accelerate localized deformation and microcrack nucleation, leading to a self-amplifying process involving hydrogen accumulation, stress concentration, and crack propagation [22]. Using molecular dynamics simulations, Song et al. [23] revealed that hydrogen enrichment at crack-tip regions enhances localized plastic deformation, while the peak stress of α-Fe decreases progressively with increasing hydrogen concentration, highlighting the concentration-dependent modification of local mechanical responses. Through cyclic loading experiments, Matsumoto et al. [24] further demonstrated that hydrogen accumulation occurs predominantly during elastic deformation and decreases near the onset of yielding, indicating a strong coupling between hydrogen redistribution and deformation evolution. Therefore, hydrogen-induced fracture cannot be adequately understood by considering stress fields or hydrogen concentration independently. A comprehensive understanding requires a coupled analysis integrating hydrogen transport, stress evolution, and microstructural characteristics to reveal the governing factors controlling hydrogen embrittlement behavior.

In recent years, the critical hydrogen concentration has emerged as an important parameter for describing the hydrogen-induced ductile-to-brittle transition of metallic materials, providing a quantitative connection between microscale hydrogen redistribution and macroscopic fracture behavior. Therefore, accurately quantifying and predicting the critical hydrogen concentration, as well as identifying the microstructural factors governing its variation, have become central issues in hydrogen embrittlement research. Ruggieri et al. [25] established a coupled local fracture criterion incorporating critical stress and hydrogen concentration through finite element simulations combined with hydrogen transport models, enabling accurate prediction of fracture strength in hydrogen-charged high-strength steels. Priyadarshi and Balasubramaniam [26] investigated Fe_3_Al alloys and suggested that the critical hydrogen concentration was associated with hydrogen-induced structural evolution and interface weakening. Hasan et al. [27] further demonstrated that hydrogen clustering could induce local structural transformations and accelerate embrittlement. Experimental studies by Lin et al. [28] and others confirmed that brittle fracture occurs only when local hydrogen concentration exceeds a certain threshold value. With the development of multiscale modeling and experimental characterization techniques, the concept of critical hydrogen concentration has been extended to different materials, microstructural regions, and characteristic length scales. For example, Ngiam et al. [29] employed a coupled phase-field model to determine critical average hydrogen concentrations associated with embrittlement risks at lath boundaries and prior austenite grain boundaries, and proposed a fracture mode prediction approach based on the comparison between local hydrogen concentration and the corresponding critical value. Kawamori and Yuse [30] experimentally demonstrated that hydrogen embrittlement occurred when surface hydrogen concentration exceeded the critical threshold, while Xing et al. [31] revealed the relationship between hydrogen concentration and plastic-zone saturation during hydrogen-induced ductile-to-brittle transition through molecular dynamics simulations. However, most existing studies have evaluated critical hydrogen concentration under relatively fixed microstructural conditions, treating it as a material-dependent threshold without fully considering its dynamic dependence on microstructural hydrogen trapping and redistribution. In particular, the influence of grain size variation on grain-boundary hydrogen segregation, local hydrogen accumulation, and the evolution of critical hydrogen concentration remains insufficiently understood and requires systematic investigation.

In summary, although significant progress has been achieved in understanding hydrogen embrittlement mechanisms and critical hydrogen concentrations, existing studies have largely focused on isolated effects of hydrogen concentration, stress state, or microstructural characteristics at specific length scales. A comprehensive understanding of the coupled relationships among grain size, hydrogen distribution, dislocation-mediated deformation, and critical hydrogen concentration remains lacking. In particular, the evolution of hydrogen-induced dislocation behavior from mobility enhancement to suppression, as well as the underlying mechanism controlling this transition, has not been fully clarified. Furthermore, the role of grain size in regulating grain-boundary hydrogen segregation, dislocation evolution, and the hydrogen-induced ductile-to-brittle transition process remains insufficiently understood. More importantly, a physically based quantitative relationship linking grain size, grain-boundary atom fraction, and critical hydrogen concentration has yet to be established. To address these issues, molecular dynamics simulations were employed in this study using polycrystalline α-Fe models with systematically varied grain sizes. The effects of hydrogen concentration and grain size on hydrogen redistribution, dislocation evolution, and intergranular fracture behavior were investigated to reveal the microscopic mechanisms governing the hydrogen-induced ductile-to-brittle transition. It should be noted that the present simulations employ idealized polycrystalline α-Fe models and therefore do not include all microstructural complexities of practical pipeline steels, such as secondary phases, inclusions, and residual stress states. Accordingly, the predicted critical hydrogen concentration should be regarded as a model-dependent atomistic indicator, while further multiscale studies are required to incorporate additional engineering factors. Based on the identified grain-size-dependent hydrogen trapping and deformation mechanisms, a predictive approach incorporating grain size and grain-boundary atom fraction was proposed to evaluate the critical hydrogen concentration. This work provides an atomic-scale mechanistic understanding of hydrogen-induced fracture transition and offers theoretical guidance for hydrogen embrittlement assessment and structural integrity design of hydrogen transportation pipeline materials.

## 2. Methods

### 2.1. Construction of the Molecular Dynamics Model

To elucidate the microscopic deformation and fracture mechanisms of polycrystalline α-Fe under the coupled effects of hydrogen concentration and grain size, molecular dynamics simulations were performed based on constructed polycrystalline α-Fe models. Since X80 pipeline steel primarily consists of acicular ferrite at room temperature and its ferritic matrix exhibits a body-centered cubic (BCC) α-Fe structure, polycrystalline α-Fe was adopted as an atomic-scale representation of the ferritic matrix of pipeline steels. This simplified model captures the essential grain-boundary-mediated deformation characteristics of the ferritic matrix while enabling investigation of hydrogen-induced embrittlement mechanisms at the atomic scale. The polycrystalline α-Fe models were generated using the Atomsk software (Beta 0.13.1) [32]. A Voronoi tessellation method was employed to construct randomly oriented grains and introduce grain boundaries with different crystallographic orientations, providing a realistic representation of the heterogeneous microstructure of ferritic pipeline steels. To investigate the influence of grain size on hydrogen embrittlement behavior, two polycrystalline models containing 10 and 20 grains were constructed within identical simulation cells, corresponding to different average grain sizes. Both models had dimensions of 200 Å × 200 Å × 200 Å and contained 663,273 Fe atoms. Periodic boundary conditions were applied along the x, y, and z directions to eliminate free-surface effects and reproduce bulk-like mechanical behavior. All molecular dynamics simulations were performed using the LAMMPS package, while atomic configurations and dislocation evolution were visualized and analyzed using OVITO (Version 3.9.4) [33,34]. The metal unit system was adopted throughout the simulations. The systems were equilibrated under the isothermal–isobaric (NPT) ensemble to obtain stable initial configurations under controlled thermodynamic conditions before subsequent deformation simulations [35].

### 2.2. Interatomic Potentials and Model Relaxation

**Figure 1:**
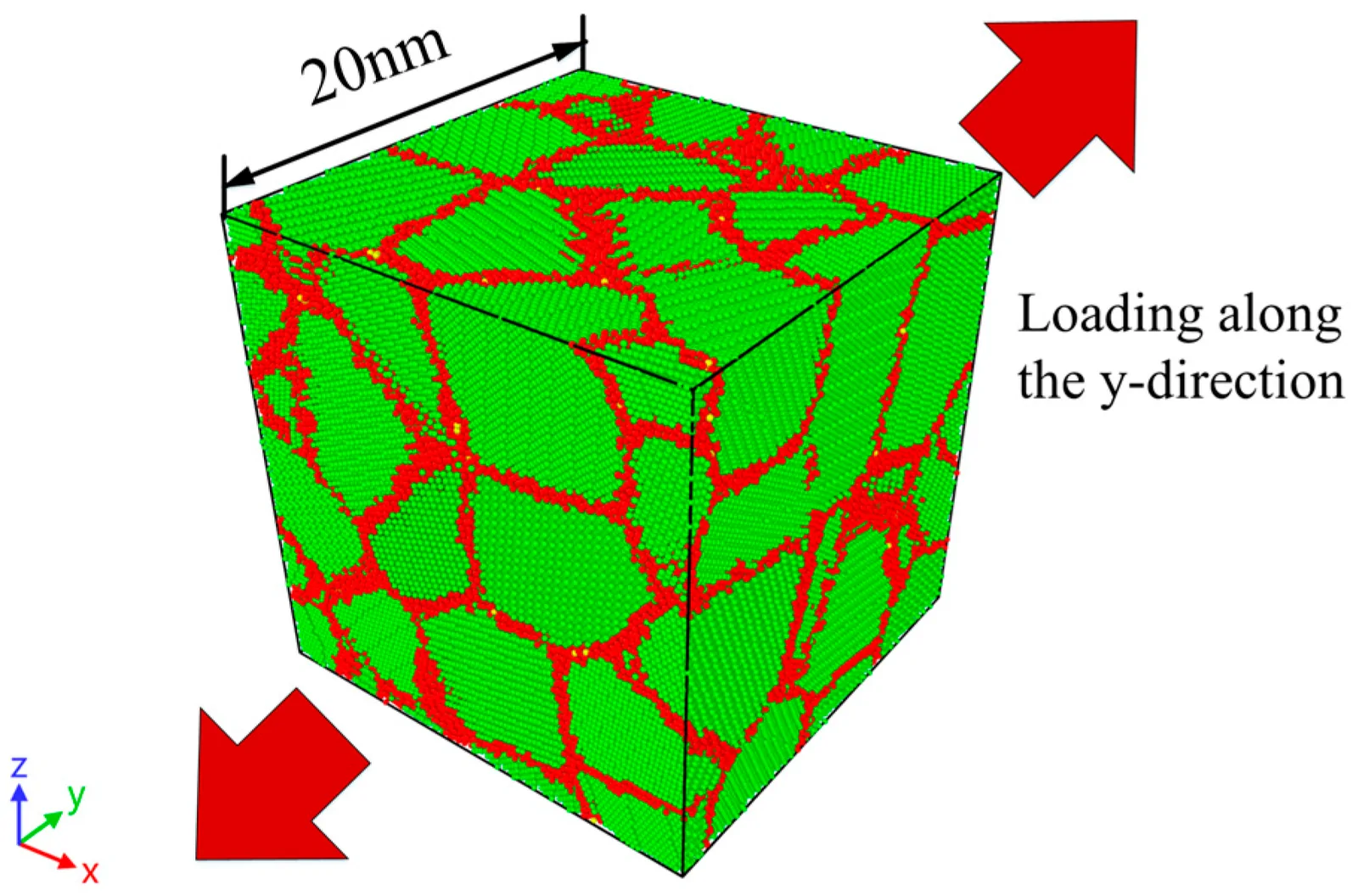
Relaxed polycrystalline α-Fe model and schematic illustration of uniaxial tensile loading. Green atoms denote BCC lattice atoms, whereas red atoms indicate grain-boundary atoms.

The selection of appropriate interatomic potentials strongly influences the accuracy of atomic interactions and is therefore essential for ensuring the reliability of molecular dynamics simulations. The embedded atom method (EAM) has been widely employed for metallic systems because it considers many-body interactions and provides reliable descriptions of lattice properties, elastic behavior, and defect evolution in BCC iron. Therefore, the EAM potential developed by Mendelev et al. was adopted to describe Fe–Fe interactions in this study [36,37]. The Fe–H interactions were described using a DFT-calibrated interaction potential, which was developed to accurately reproduce hydrogen-related behaviors in BCC Fe, including hydrogen diffusion, segregation, and trapping processes [14]. After constructing the atomic models, thermal annealing relaxation was performed to eliminate artificial high-energy configurations and promote grain-boundary reconstruction and stress relaxation. Specifically, the models were initially heated from 300 K to 700 K at a heating rate of 160 K/ns over 2.5 ns. The systems were then maintained at 700 K for an additional 2.5 ns to allow sufficient structural relaxation. Subsequently, the systems were cooled to 300 K at the same cooling rate and further equilibrated for 2.5 ns to obtain stable polycrystalline α-Fe structures. The relaxed polycrystalline α-Fe model is presented in Figure 1.

### 2.3. Hydrogen Introduction and Diffusion

After structural relaxation, hydrogen atoms were introduced into the polycrystalline α-Fe models according to the prescribed hydrogen concentrations. Hydrogen concentrations of 0.5, 1.0, 1.5, 2.0, and 2.5 at.% were considered to investigate the deformation and fracture behavior under different hydrogen charging conditions. The hydrogen concentration was defined as the atomic ratio of hydrogen atoms to iron atoms (H/Fe). Since tetrahedral interstitial sites are energetically favorable positions for hydrogen occupation in BCC α-Fe, hydrogen atoms were initially randomly distributed among available tetrahedral sites to represent the initial hydrogen incorporation state in the lattice. The initial hydrogen distribution was not determined according to an experimental diffusion profile, and hydrogen atoms were not artificially assigned to grain boundaries or other specific microstructural regions. After hydrogen insertion, the systems were further equilibrated under the NPT ensemble to allow hydrogen diffusion, lattice relaxation, and redistribution toward energetically favorable trapping sites. During this equilibration process, hydrogen atoms migrated from their initial random positions and interacted with microstructural features, such as grain boundaries. The evolution of the atomic configuration was evaluated, and the equilibrated structures obtained after hydrogen redistribution were subsequently employed as the initial structures for tensile deformation simulations. The equilibration process was continued until the structural energy and hydrogen distribution reached a stable state. The grain-size-dependent hydrogen segregation behavior at grain boundaries and within grain interiors was then analyzed. The equilibrated configurations were subsequently employed as the initial structures for tensile deformation simulations.

### 2.4. Uniaxial Tensile Simulation

After hydrogen redistribution reached equilibrium, uniaxial tensile loading was applied to investigate the deformation and fracture behavior of polycrystalline α-Fe models with different hydrogen concentrations and grain sizes. Tensile deformation was performed along the y-direction, while periodic boundary conditions were maintained along the x- and z-directions to minimize artificial surface effects. A constant strain rate of 0.1 ns^−1^ was applied during the tensile simulations, with a total deformation duration of 3 ns. This strain rate was selected considering the intrinsic timescale limitation of molecular dynamics simulations. Due to the limited timescale accessible in atomistic simulations, relatively high strain rates are generally required to investigate deformation and fracture processes within a computationally feasible period. Although this strain rate is considerably higher than those used in conventional experimental tensile tests, it enables comparative analysis of atomic-scale deformation mechanisms, including hydrogen redistribution, dislocation evolution, and crack initiation, under identical loading conditions. Therefore, the present study focuses on the relative effects of hydrogen concentration and grain size on fracture behavior rather than directly reproducing experimental strain-rate-dependent mechanical responses. During deformation, the stress–strain response, dislocation evolution, crack initiation and propagation behavior, and hydrogen redistribution were continuously monitored. The obtained structural and mechanical responses were analyzed to reveal the grain-size-dependent hydrogen-induced ductile-to-brittle transition mechanisms in polycrystalline α-Fe. The critical hydrogen concentration was subsequently evaluated based on the fracture transition behavior under different grain-size conditions.

## 3. Results and Discussion

### 3.1. Effect of Hydrogen Concentration on Hydrogen Diffusion

In the hydrogen-charged models, hydrogen atoms were initially inserted randomly into tetrahedral interstitial sites and distributed throughout the model. After 2 ns of diffusion, the hydrogen distribution gradually approached a stable state. During the diffusion process, grain boundaries acted as the primary transport pathways for hydrogen atoms, and most hydrogen atoms ultimately accumulated and became stabilized at the grain boundaries, indicating that grain boundaries exerted a pronounced trapping effect on hydrogen. To quantitatively characterize the diffusion behavior of hydrogen atoms, the mean squared displacement (MSD) was calculated using Equation (1) [3].

(1) $$MSD=\frac{1}{2dN}\sum_{1}^{N}{\left|r_{i}\left(t_{0}+\Delta t\right)-r_{i}\left(t_{0}\right)\right|}^{2}$$

where *d* is the dimensionality of the model system, which is 3 in the present study; *N* is the total number of hydrogen atoms; *r_i_*(*t*_0_) is the initial position of the *i*-th hydrogen atom; and *r_i_* (*t*_0_ + Δt) is the position of the *i*-th hydrogen atom after a time interval of Δ*t*.

**Figure 2:**
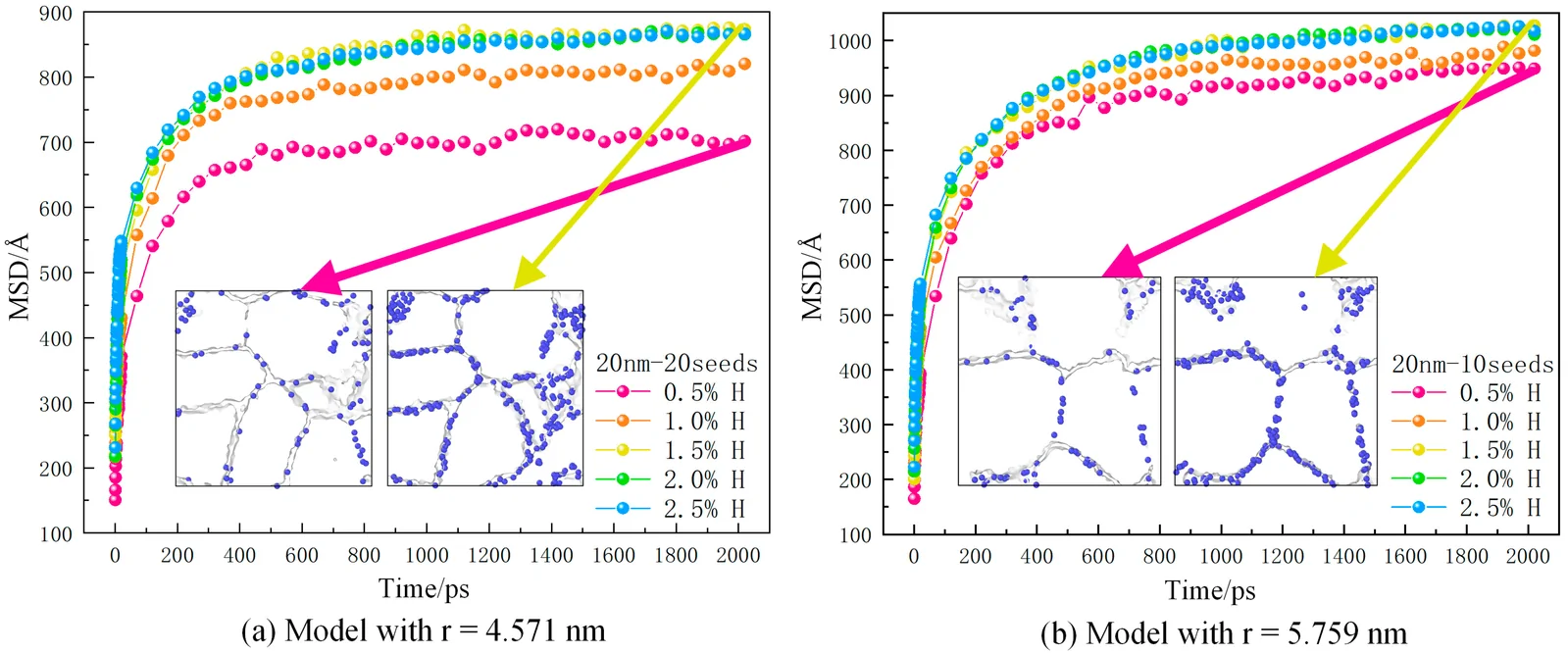
MSD curves of models with different hydrogen concentrations.

The variations in the MSD values of the 10-grain and 20-grain models at different hydrogen concentrations during diffusion are shown in Figure 2. Within the first 100 ps, the MSD of hydrogen atoms increased markedly, corresponding to a stage of rapid diffusion. As the hydrogen concentration increased, the MSD values became larger, and the slopes of the MSD curves became steeper, indicating that the concentration gradient played an important role in driving hydrogen diffusion. In the later stage of diffusion, the variation in MSD gradually decreased, and the diffusion process progressively leveled off. At this stage, hydrogen atoms were largely confined by the grain boundaries and gradually reached an energetically stable state. Under stabilized diffusion conditions, the total MSD first increased with increasing hydrogen concentration and then tended to saturate, with a slight decrease even observed in the range of 1.5–2.0%. This behavior indicates that the internal space within the grain-boundary pathways gradually approached saturation, thereby reducing the space available for hydrogen migration and weakening the concentration gradients between different regions. As a result, the driving force for hydrogen migration gradually approached saturation. Therefore, 1.5% was identified as the critical concentration at which the concentration-gradient-driven hydrogen diffusion capacity within the grain boundaries began to approach saturation.

For models with the same overall dimensions but different numbers of grains, the 10-grain model corresponds to a coarse-grained structure. Owing to its lower grain-boundary fraction, the grain-boundary network in this model is relatively simple and contains fewer tortuous paths. Consequently, the MSD of hydrogen atoms is consistently higher at all hydrogen concentrations. Under diffusion-stabilized conditions, the total MSD of the 10-grain coarse-grained model is approximately 13% higher than that of the 20-grain fine-grained model. Nevertheless, the overall variation pattern and trend remain consistent with those observed in the fine-grained model.

### 3.2. Effect of Hydrogen Concentration on Dislocation Nucleation and Emission

**Figure 3:**
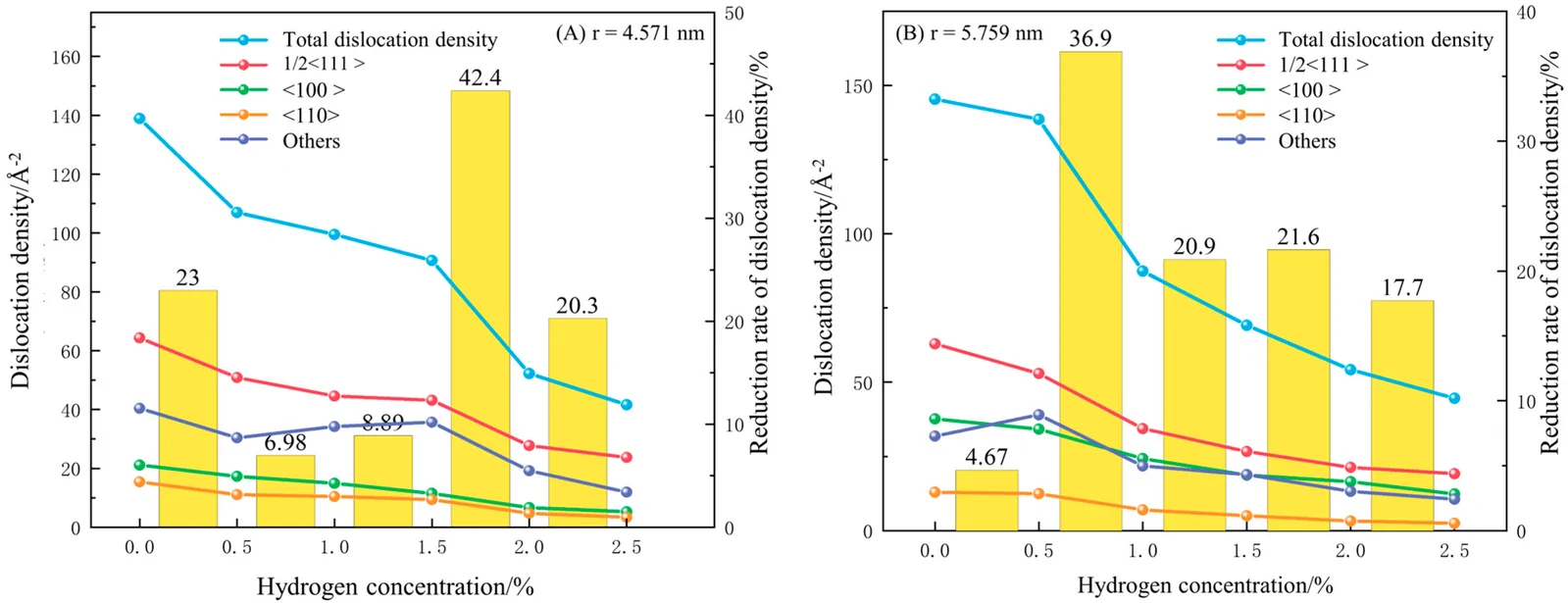
Comparison of total dislocation density evolution during tensile deformation at different hydrogen concentrations for the fine-grained and coarse-grained models.

The dislocation evolution during tensile deformation was analyzed for models with hydrogen-to-iron atomic ratios ranging from 0% to 2.5%. Because the present models have a BCC structure, the dislocations were classified according to their Burgers vectors into 1/2<111>, <100>, <110>, and other types. The total dislocation density was calculated as the ratio of the total dislocation line length to the model volume, and the values were recorded at intervals of 0.2% strain during tensile deformation. The detailed evolution of different dislocation types for the two grain-size models is provided in Appendix A, while the comparison of the total dislocation density evolution is presented in Figure 3.

In both models with different numbers of grains, the dominant dislocation type is 1/2<111>, which is consistent with previous studies [38]. With increasing hydrogen concentration, the densities of all dislocation types decrease noticeably, suggesting that hydrogen suppresses dislocation nucleation and emission within a certain concentration range. Furthermore, when the hydrogen concentration exceeds 1.5%, the densities of all dislocation types become significantly lower than those in the hydrogen-free model and then remain nearly stable. This behavior is particularly evident prior to crack initiation, when the internal structure of the model remains relatively intact, and energy is released entirely through internal structural transformations. Therefore, increasing hydrogen concentration alters the pathway of energy dissipation, further supporting the identification of 1.5% as the critical hydrogen concentration at which the fracture mechanism transitions under the present conditions.

### 3.3. Effect of Hydrogen Concentration on Fracture Initiation

**Figure 4:**
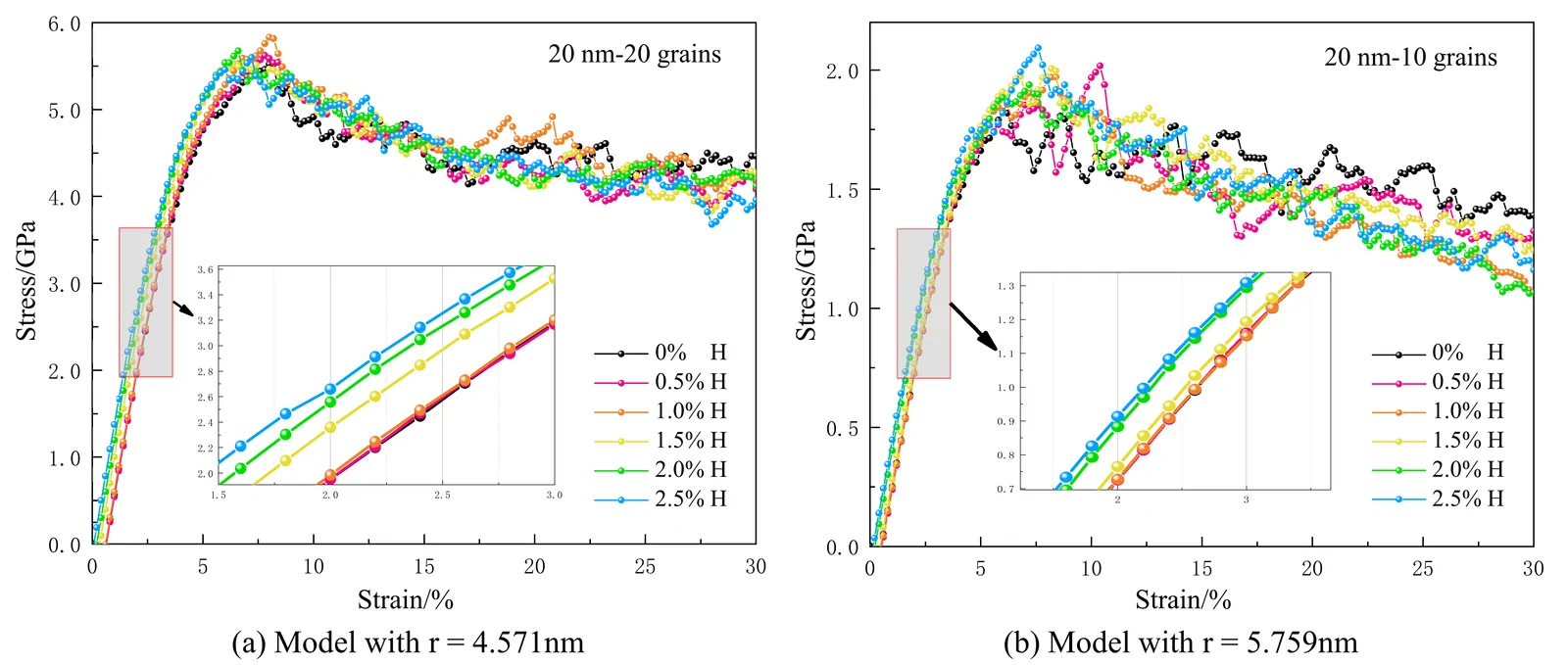
Stress–strain curves of models with different hydrogen concentrations.

The results obtained from LAMMPS were analyzed on a frame-by-frame basis. The stress evolution during tensile deformation was determined by summing and averaging the stress components of all atoms along the loading direction. For each frame, the corresponding strain was calculated from the prescribed strain rate and the total loading time. The stress and strain data were then combined to construct the stress–strain curves under the given conditions. The stress–strain curves were further integrated to evaluate the variation in fracture energy. The stress–strain responses of models with hydrogen-to-iron atomic ratios ranging from 0 to 2.5% during tensile deformation are shown in Figure 4, in which the stress direction is parallel to the loading direction.

For the model with a side length of 20 nm containing 20 grains (*r* = 4.571 nm), the corresponding stress–strain curves are shown in Figure 4a. In the elastic stage, the slopes of the curves at different hydrogen concentrations are essentially identical, indicating that hydrogen has a negligible influence on the elastic modulus. A magnified view of the curves further shows that, at the same strain, the stress gradually increases with increasing hydrogen concentration, suggesting that hydrogen incorporation may modify the local atomic interactions, lattice distortion, and internal stress state without substantially changing the apparent elastic modulus. In the plastic stage, both the yield stress and the yield strain initially increase with increasing hydrogen concentration. However, once the hydrogen concentration exceeds 1.5%, these values tend to stabilize within a certain range and even show a slight decreasing trend. This suggests that, at relatively low concentrations, hydrogen may influence the local deformation behavior; however, the present results do not directly demonstrate enhanced plastic deformation. At higher concentrations, however, this effect gradually weakens, and the deformation capacity of the material correspondingly decreases.

For the model with a side length of 20 nm containing 10 grains (*r* = 5.759 nm), the corresponding curves are shown in Figure 4b. The influence of hydrogen is generally consistent with that observed in the 20-grain model. In the elastic stage, the elastic modulus remains largely unaffected by hydrogen concentration, whereas the stress increases noticeably when the hydrogen concentration exceeds 1.5%. However, changing the grain size in the present Voronoi-based models also modifies the grain-boundary network, triple-junction distribution, local atomic configurations, and local stress fields, which may influence crack initiation and propagation during fracture. Under these conditions, the role of grain boundaries in accommodating and dissipating deformation energy becomes more evident. For the coarse-grained model, the fracture stress is lower than that of the fine-grained model under all hydrogen concentrations, and the calculated stress values exhibit a relatively small variation range. This difference may be associated with the different grain-boundary configurations and local stress distributions generated in the two models. Meanwhile, internal cracks are predominantly large and continuous, which leads to greater scatter in the numerical results and more pronounced fluctuations in the curves. Overall, the stress-bearing capacity shows a general decreasing trend with increasing hydrogen concentration.

**Figure 5:**
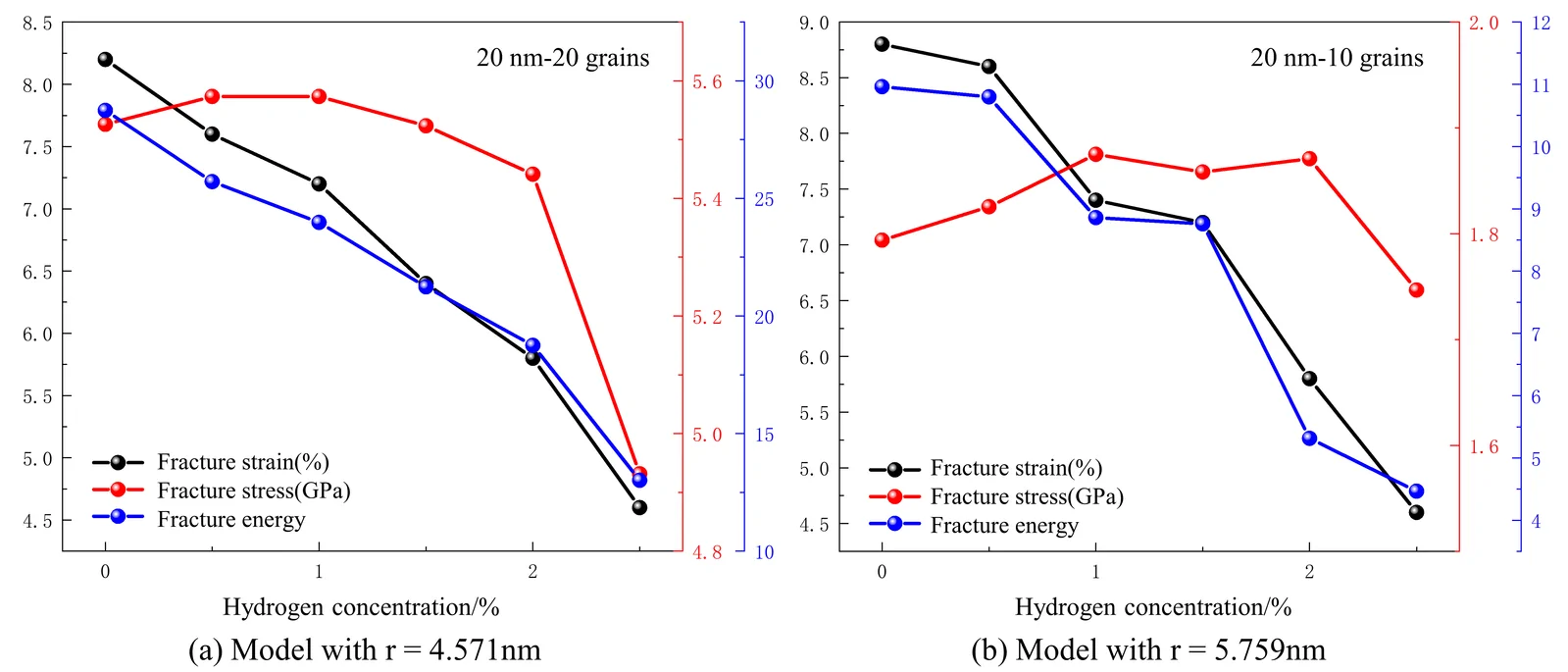
Variations in initial fracture parameters under different hydrogen concentrations.

During tensile deformation, the area enclosed by the generated stress–strain curve and the coordinate axes was taken as the fracture energy of the model. By integrating the stress–strain curves, the corresponding fracture energy values and their variations under six hydrogen concentrations ranging from 0 to 2.5% were obtained, as detailed in Table 1 and Figure 5. For all models with different grain numbers, the fracture energy exhibited a pronounced decreasing trend with increasing hydrogen concentration. Specifically, for the model with a side length of 20 nm containing 20 grains (r = 4.571 nm), the fracture energy decreased by approximately 54.72%, whereas for the model with a side length of 20 nm containing 10 grains, the corresponding decrease was approximately 59.24%; in both cases, the reduction exceeded 50%. Hydrogen markedly reduced the fracture energy of the material, indicating that the same material can sustain less energy in a hydrogen environment and is therefore more susceptible to fracture. Under the same variation in hydrogen concentration, the energy that could be sustained by the coarse-grained model was approximately one-third of that sustained by the fine-grained model, further confirming that grain boundaries serve as important buffering structures during material deformation. Because only one representative configuration was considered for each grain-size model, the individual contributions of grain size and configuration-dependent structural variations to the observed fracture-energy and fracture-stress differences cannot be independently quantified.

**Figure 6:**
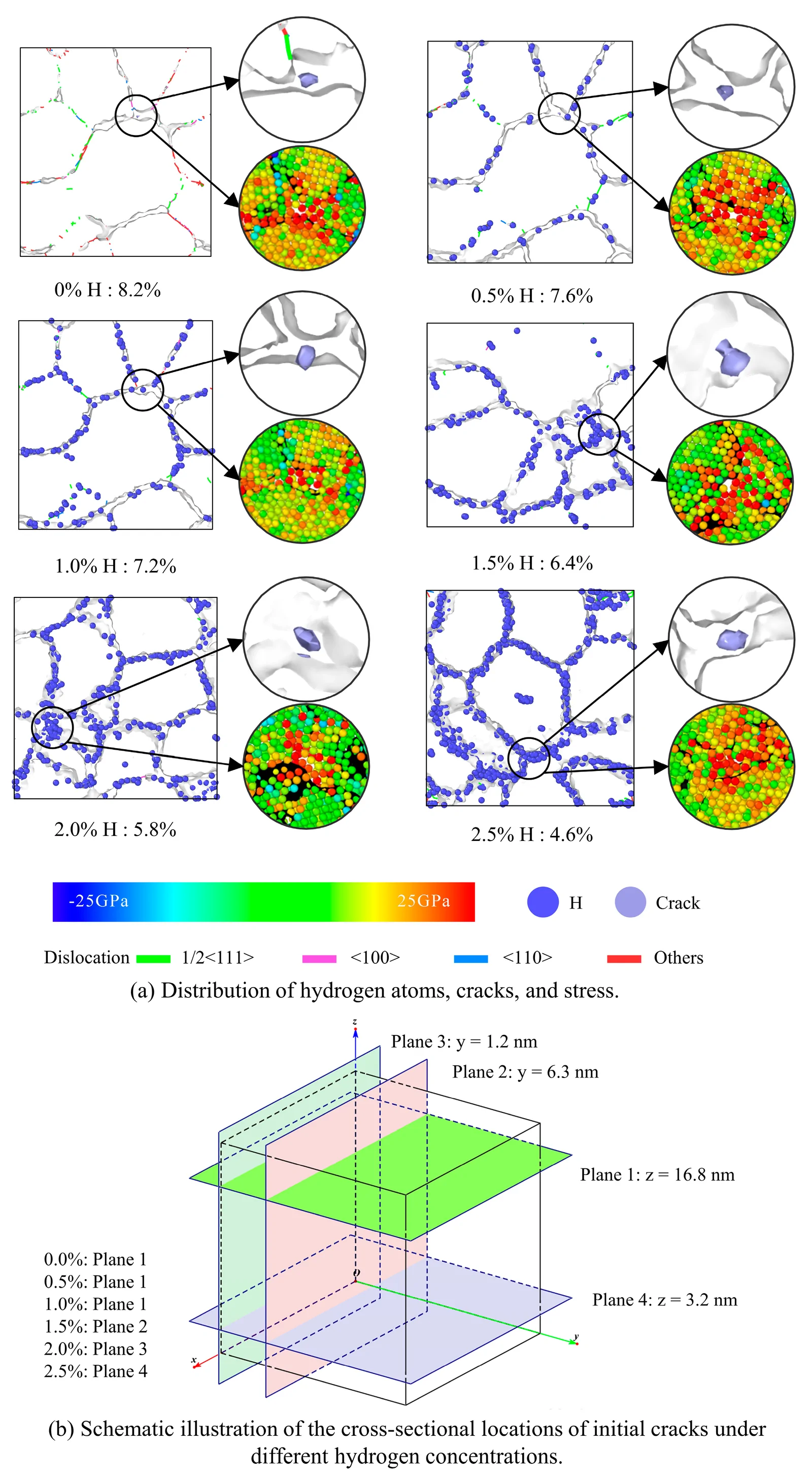
Initial fracture behavior of the model with r = 4.571 nm under different hydrogen concentrations.

For the model containing 20 grains (*r* = 4.571 nm), the strain and stress corresponding to the onset of fracture under hydrogen concentrations ranging from 0 to 2.5% are presented in Table 1 and Figure 5. With increasing hydrogen concentration, the initial fracture strain of the model decreases progressively, indicating a reduction in material ductility. Specifically, the initial fracture strain decreases by more than 40%, while the fracture energy decreases by more than 50%. By contrast, the initial fracture stress exhibits a trend of first increasing and then decreasing. To further analyze the local structural characteristics associated with fracture initiation, a slice with a thickness of 5 Å was extracted from the initial fracture location in the 20-grain model at each hydrogen concentration, as shown in Figure 6.

When the hydrogen concentration is lower than 1.5%, the location of initial crack formation remains the same as that in the hydrogen-free model under tensile loading. This site is a multi-junction grain-boundary region, where stress is relatively concentrated, and fracture is therefore more likely to occur. Before crack initiation, a large number of dislocations are emitted within a 25 Å-thick region surrounding the crack. As microscopic defects that emerge prior to fracture, dislocations can dissipate part of the substantial energy accumulated within the material during their emission, thereby extending the energy threshold required for fracture initiation. This is reflected macroscopically in a slight increase in the initial fracture stress of the model. It can thus be inferred that, at relatively low hydrogen concentrations, dislocation-mediated plastic deformation remains active near the crack-initiation region, providing local plastic accommodation before fracture. This behavior represents a HELP-like contribution; however, it does not necessarily correspond to an increase in the overall dislocation density, as the total dislocation density decreases with increasing hydrogen concentration under the present simulation conditions. With further hydrogen accumulation, the contribution of grain-boundary weakening becomes increasingly significant, leading to a gradual shift toward a HEDE-like fracture response.

When the hydrogen concentration exceeds 1.5%, the location of initial crack formation changes continuously, but it always occurs at a multi-junction grain-boundary site where both hydrogen and stress are relatively concentrated. Before crack initiation, no obvious dislocations are observed within the 25 Å-thick region surrounding the crack. A large number of hydrogen atoms within this region reduce the contribution of dislocation-mediated plastic accommodation, resulting in a more localized fracture process. The increased hydrogen accumulation near grain boundaries and the enhanced tendency for intergranular crack initiation suggest that hydrogen-assisted grain-boundary weakening becomes increasingly important at higher hydrogen concentrations. As a consequence, both the fracture energy and the fracture stress decrease.

Therefore, the results suggest that increasing hydrogen concentration changes the balance between dislocation-mediated plastic deformation and hydrogen-assisted grain-boundary fracture. At relatively low hydrogen concentrations, hydrogen promotes local plastic accommodation through enhanced dislocation activity. With further hydrogen enrichment, however, reduced dislocation-mediated deformation and increased grain-boundary fracture characteristics become dominant, resulting in a gradual transition toward a more brittle fracture response.

### 3.4. Effect of Hydrogen Concentration on Crack Propagation

**Figure 7:**
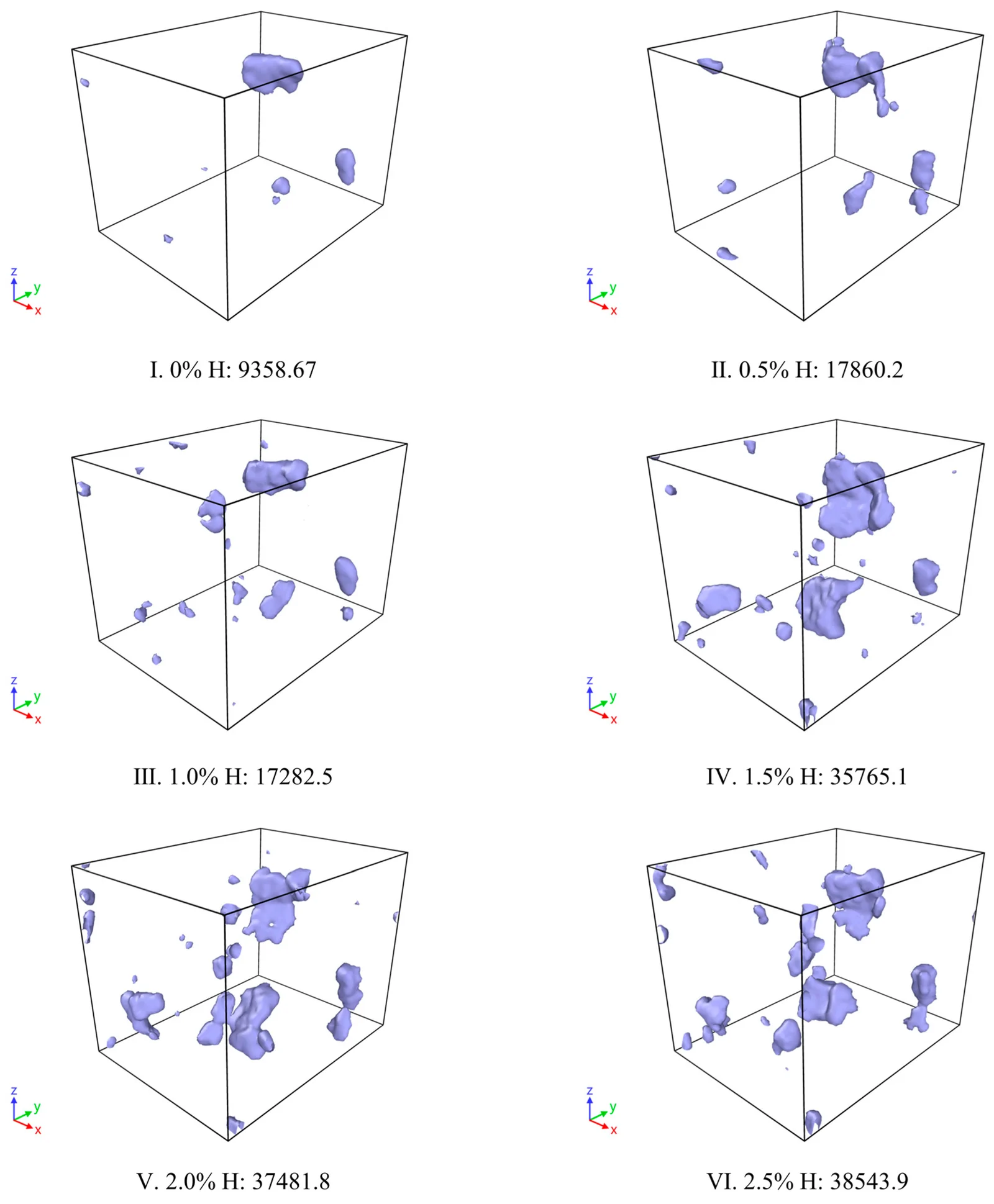
Defects and crack surface area of the model with r = 4.571 nm at 20% strain.

In the present study, the crack distributions in all models were examined at strains of 10%, 20%, and 30% under all hydrogen concentrations. As shown in Figure 7, the crack distribution at 20% strain is presented as a representative example. At the same strain level, both the number and the surface area of cracks increase progressively with increasing hydrogen concentration. When the crack volumes are of the same order of magnitude, the variation trend of crack volume is consistent with that of crack surface area; that is, the crack volume also increases with increasing hydrogen concentration.

**Figure 8:**
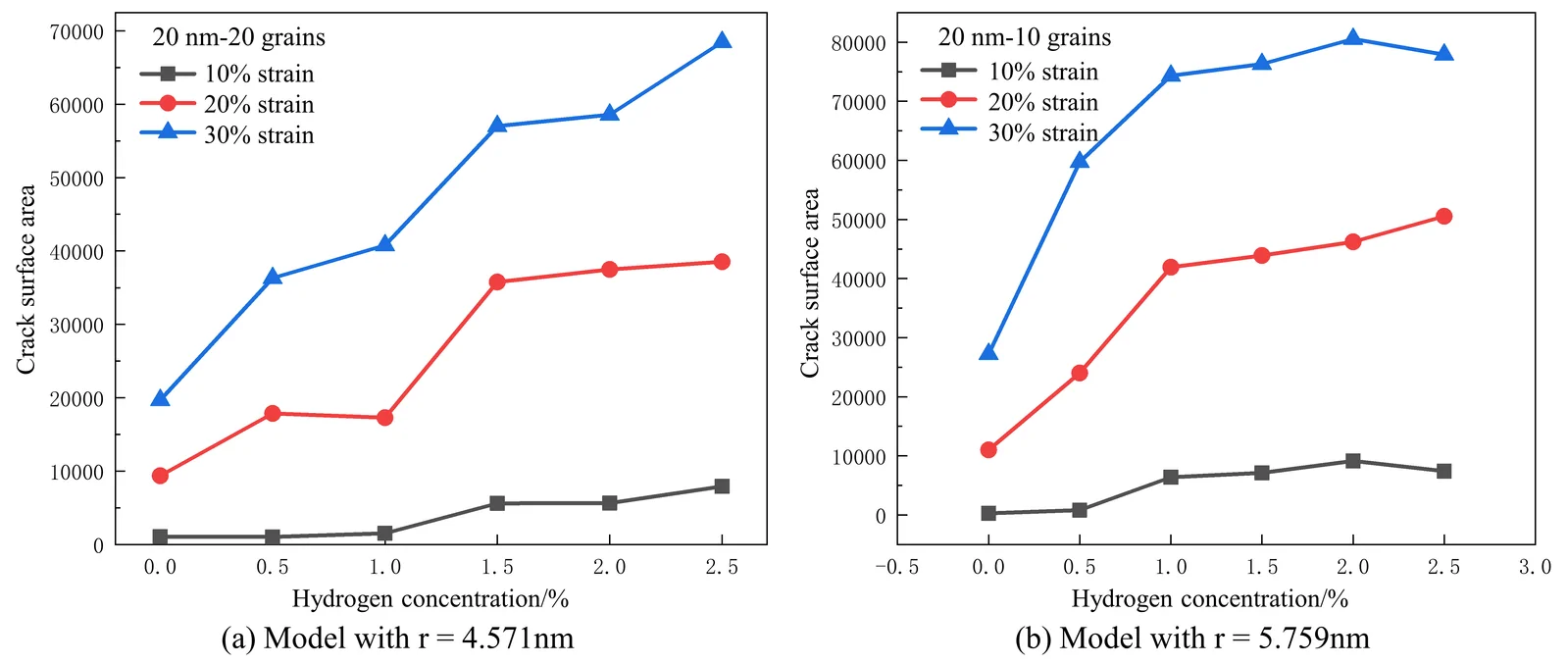
Variation trends of crack surface area of the models at different strain levels.

The crack surface area was quantified, as summarized in Table 2 and Figure 8. With increasing hydrogen concentration, not only is the threshold for crack initiation reduced, but crack propagation is also promoted. For the model with a side length of 20 nm containing 20 grains (*r* = 4.571 nm), a distinct step-like increase in crack surface area is observed within the hydrogen concentration range of 1.0–1.5% at all three strain levels, which further supports the previously inferred critical concentration. When the hydrogen concentration exceeds 1.5%, the role of hydrogen in dislocation behavior within the material shifts from promotion to suppression. Without the buffering effect provided by dislocation-mediated energy dissipation, more fracture sites are generated. Meanwhile, plastic zones develop at the edges of existing cracks, inhibiting crack-tip blunting and thereby promoting the formation of cracks with larger volumes.

For the model with a side length of 20 nm containing 10 grains (*r* = 5.759 nm), the cracks are relatively more continuous, resulting in a larger overall crack surface area, which is consistent with the previously obtained stress results. Owing to the inherent stochastic variability of the simulation and the discrete nature of the selected hydrogen concentrations, a turning point in the growth trend of crack surface area is observed within the concentration range of 1.0–1.5%. Nevertheless, from the overall trend, the crack surface area increases rapidly when the hydrogen concentration is below 1.0%, whereas the growth becomes relatively gradual when the concentration exceeds 1.5%. This indicates that the critical transition concentration lies approximately within the range of 1.0–1.5%, which is consistent with the conclusions presented earlier.

During the application of 30% strain over the entire range of hydrogen concentrations, the crack surface area of the model containing 20 grains increased by a factor of 2.48, while that of the model containing 10 grains increased by 1.86 times. This indicates that, since the primary effect of hydrogen embrittlement occurs at grain boundaries, the fine-grained model exhibits higher sensitivity to hydrogen embrittlement. Although the coarse-grained model shows a smaller relative change before and after deformation, the crack surface area generated under the same strain is approximately 1.2 times that of the fine-grained model. This suggests that grain boundaries serve as an important mechanism for strain accommodation in materials, resulting in reduced damage in fine-grained models under identical loading conditions.

### 3.5. Predictive Relationship Between Grain Size and Critical Hydrogen Concentration

For the model with a side length of 20 nm containing 20 grains, the critical hydrogen-to-iron atomic ratio is 1.5%, and the fraction of grain-boundary atoms is 20.3%. Under these conditions, the corresponding ratio of hydrogen atoms to grain-boundary atoms at the transition of the hydrogen effect is 7.39%. For the model with a side length of 20 nm containing 10 grains, the critical hydrogen-to-iron atomic ratio is also 1.5%, while the fraction of grain-boundary atoms is 18.3%, corresponding to a hydrogen-to-grain-boundary atom ratio of 8.20% at the transition point. Considering that the number of hydrogen atoms used in the calculations is discrete rather than continuous, it may be reasonably inferred that, for the same material, the critical grain-boundary hydrogen concentration associated with the transition of the hydrogen embrittlement mechanism lies within the range of 7–9%.

**Figure 9:**
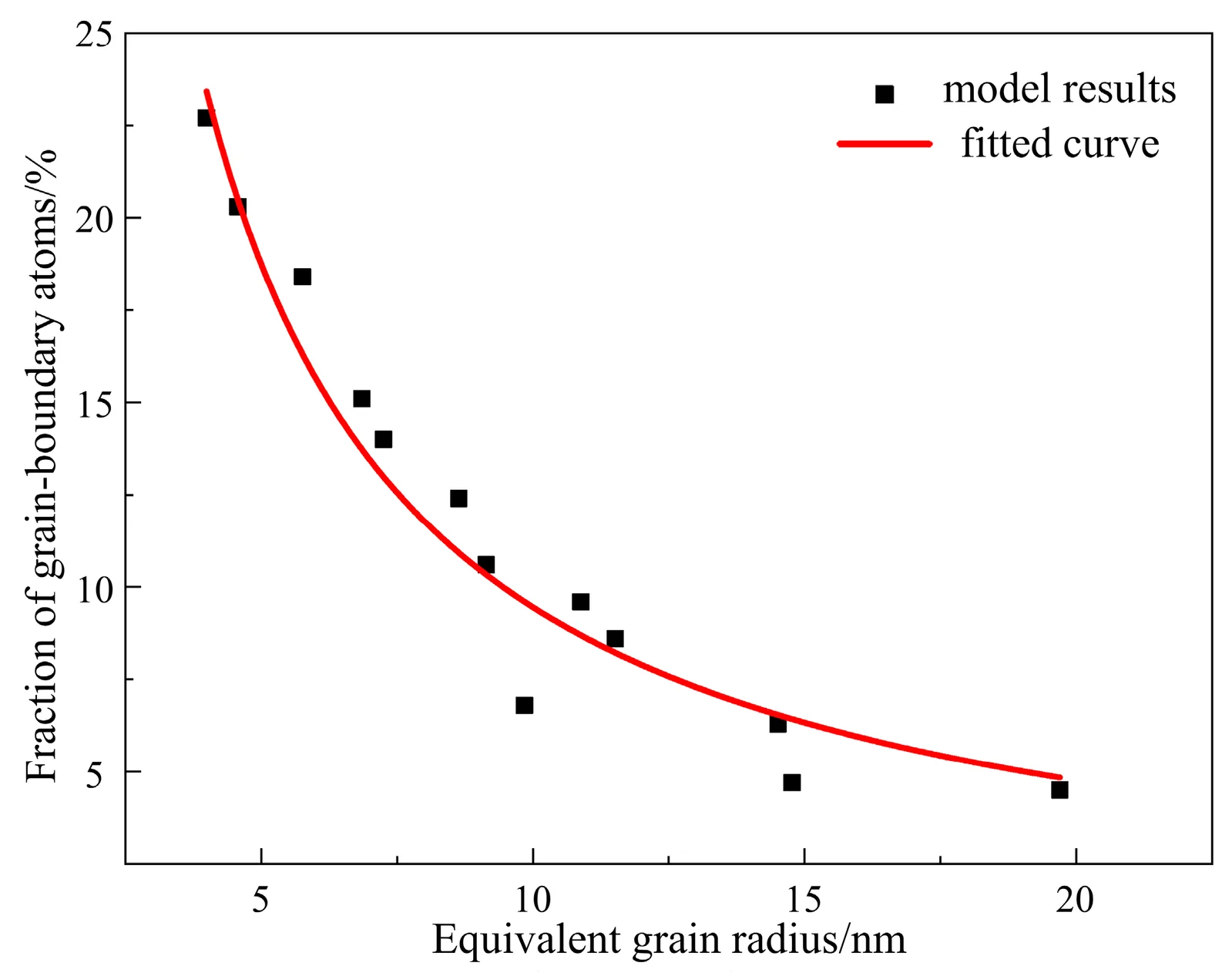
Relationship between equivalent grain radius and fraction of grain-boundary atoms.

After diffusion, most hydrogen atoms are concentrated at the grain boundaries, and the observed changes in mechanical behavior are closely related to the gradual saturation of hydrogen concentration within these regions. Therefore, to further establish the relationship between microstructural scale and critical hydrogen concentration, energy minimization was performed on models with different sizes and grain numbers. Based on these models, the relationship between the equivalent grain radius and the fraction of grain-boundary atoms was obtained, as shown in Table 3 and Figure 9.

By numerical fitting, a power-law relationship between the equivalent grain radius and the fraction of grain-boundary atoms was obtained as follows:

(2) $$p=91.97755r^{-0.98807}$$

where *p* denotes the fraction of grain-boundary atoms, with *p* = *a*_boundary_/*a*_grain_, *a*_boundary_ and *a*_grain_ represent the number of grain-boundary atoms and the total number of atoms in the grain, respectively; and *r* is the equivalent grain radius.

Regression analysis yielded an R^2^ value of 1, indicating excellent agreement between the fitted relationship and the calculated data. This relationship may therefore be used to estimate the fraction of grain-boundary atoms in polycrystalline α-Fe structures with different grain sizes under comparable model conditions.

In summary, once the grain size of a given material is known, the fraction of grain-boundary atoms can be calculated from the fitted curve. By further combining this result with the finding that the critical grain-boundary hydrogen concentration lies within the range of 7–9%, the critical hydrogen concentration of that material for a model of a given size can be approximately estimated. However, it should be noted that this estimated critical hydrogen concentration is obtained under the assumptions of the present atomistic models. In practical materials, hydrogen diffusion kinetics, environmental conditions, stress states, and microstructural characteristics may influence the actual hydrogen distribution and the critical hydrogen concentration required for fracture initiation. Future work will focus on experimental validation under continuously varying hydrogen concentrations and more realistic microstructural conditions, thereby further improving the applicability of this atomistic framework for investigating hydrogen embrittlement mechanisms.

### 3.6. Discussion

It should be emphasized that the observed ductile-to-brittle transition does not represent a complete transformation from HELP to HEDE. In practical hydrogen embrittlement processes, these two mechanisms may coexist and compete, and their relative contributions depend on hydrogen concentration, local stress state, and microstructural characteristics. In the present simulations, the continued dislocation-mediated plastic deformation at relatively low hydrogen concentrations suggests a HELP-like contribution by providing local plastic accommodation and energy dissipation before fracture. In contrast, the preferential hydrogen accumulation at grain boundaries and the increased tendency for intergranular fracture under high hydrogen concentrations indicate an increasing contribution from HEDE-like grain-boundary weakening. Therefore, the fracture evolution observed in this study is better described as a gradual shift in the balance between hydrogen-assisted plasticity and hydrogen-assisted grain-boundary degradation, rather than a simple transition between two independent mechanisms.

Although the present simulations provide insights into the hydrogen-induced fracture behavior of polycrystalline α-Fe, several limitations should be acknowledged. The current models were constructed using the Voronoi tessellation method and subsequently subjected to thermal annealing and structural relaxation before hydrogen introduction. Therefore, the simulated grain boundaries are not completely ideal geometrical interfaces, as local atomic rearrangement, grain-boundary reconstruction, and stress relaxation occur during the equilibration process. However, practical pipeline steels contain more complex microstructural features, including pre-existing vacancies, dislocation networks, inclusions, residual stress fields, and processing-induced grain-boundary defects. These features may influence hydrogen trapping behavior, diffusion pathways, local stress distribution, and crack initiation processes. Such defect structures were not explicitly incorporated into the present models. Therefore, the conclusions obtained in this work primarily describe the intrinsic coupling effects of hydrogen redistribution, grain size, and grain-boundary-mediated deformation in relaxed polycrystalline α-Fe structures, rather than fully reproducing the complex hydrogen embrittlement behavior of engineering pipeline steels.

It should also be noted that the present atomistic model has not yet been directly validated against experimental measurements. The primary objective of this work is to elucidate the fundamental mechanisms governing hydrogen-induced deformation and fracture in polycrystalline α-Fe, rather than to quantitatively reproduce the mechanical response of practical pipeline steels. Future studies combining experimental validation, realistic microstructural reconstruction, and multiscale modeling approaches will be necessary to further improve the predictive capability and engineering applicability of atomistic simulations for hydrogen embrittlement. Such efforts will provide a more comprehensive understanding of the synergistic effects of hydrogen, microstructural heterogeneity, and pre-existing defects on crack initiation and propagation in pipeline steels.

## 4. Conclusions

Molecular dynamics simulations were performed to systematically investigate hydrogen diffusion, dislocation evolution, and tensile fracture behavior in polycrystalline α-Fe with different grain sizes under various hydrogen concentrations. The underlying mechanisms governing the hydrogen-induced ductile-to-brittle transition were clarified by correlating hydrogen distribution, dislocation activity, and fracture evolution. Furthermore, a quantitative relationship among grain size, grain-boundary atom fraction, and critical hydrogen concentration was established, providing a grain-size-dependent approach for predicting the onset of hydrogen-induced ductile-to-brittle transition. The main conclusions are summarized as follows.

(1) Hydrogen diffusion and segregation behavior were primarily governed by grain-boundary trapping, which played a critical role in initiating hydrogen-induced embrittlement. During hydrogen diffusion, the MSD of hydrogen atoms gradually increased and subsequently approached a steady state as hydrogen atoms were progressively captured by grain boundaries. After reaching diffusion equilibrium, the MSD increased with increasing hydrogen concentration and eventually reached saturation, indicating the gradual exhaustion of available grain-boundary trapping sites. The saturation behavior suggests that the critical bulk hydrogen concentration corresponding to grain-boundary trap saturation is approximately 1.5%.

(2) Hydrogen concentration significantly influenced the plastic deformation capability of polycrystalline α-Fe by regulating dislocation emission and evolution. The 1/2<111> dislocations were identified as the dominant carriers of plastic deformation during tensile loading. Increasing hydrogen concentration resulted in a continuous reduction in the densities of various dislocation types, demonstrating that hydrogen accumulation suppressed dislocation activity and hindered dislocation nucleation and propagation. When the hydrogen concentration exceeded approximately 1.5%, dislocation emission was substantially inhibited, leading to reductions of more than 40% in fracture strain and more than 50% in fracture energy.

(3) Hydrogen concentration determined the crack initiation behavior and fracture mechanism transition, thereby controlling the critical conditions for hydrogen-induced ductile-to-brittle transition. At hydrogen concentrations below the critical value, crack initiation occurred at multi-junction grain-boundary regions with high stress concentration, similar to the hydrogen-free condition, and fracture remained dominated by plastic deformation. Once the critical concentration was exceeded, hydrogen accumulation at grain boundaries suppressed dislocation emission and promoted intergranular fracture. As a result, the dominant fracture mechanism gradually transitioned from HELP to HEDE. The fracture surface areas increased by factors of 2.48 and 1.86 for the fine-grained and coarse-grained models, respectively, indicating that the fine-grained model exhibited a higher susceptibility to hydrogen-induced degradation due to its larger grain-boundary fraction, despite its enhanced deformation capability.

(4) A quantitative correlation among grain size, grain-boundary atom fraction, and critical hydrogen concentration was established. By fitting the relationship between equivalent grain radius and grain-boundary atom fraction, the grain-boundary structural characteristics were quantitatively predicted from grain size. Combined with the identified critical grain-boundary hydrogen concentration range of approximately 7–9 at.%, a grain-size-dependent prediction method was developed for determining the critical hydrogen concentration associated with hydrogen-induced ductile-to-brittle transition. This atomistic mechanism-based approach provides a theoretical framework for evaluating hydrogen embrittlement susceptibility and optimizing microstructural design strategies for materials with different grain sizes.

## Figures and Tables

**Table 1:** Initial fracture parameters under different hydrogen concentrations.

Model	Hydrogen Concentration/%	Initial Fracture Strain/%	Initial Fracture Stress/GPa	Fracture Energy
r = 4.571 nm	0	8.2	5.52599	28.742
	0.5	7.6	5.57337	25.713
	1.0	7.2	5.57346	23.982
	1.5	6.4	5.52360	21.241
	2.0	5.8	5.44114	18.753
	2.5	4.6	4.93156	13.013
r = 5.759 nm	0	8.8	1.79385	10.960
	0.5	8.6	1.82547	10.796
	1.0	7.4	1.87496	8.858
	1.5	7.2	1.85826	8.758
	2.0	5.8	1.87081	5.314
	2.5	4.6	1.74683	4.467

**Table 2:** Crack surface area of the models at different strain levels.

Model	Hydrogen Concentration/%	10% Strain	20% Strain	30% Strain
r = 4.571 nm	0	1061.65	9358.67	19,692.9
	0.5	1029.82	17,860.2	36,315.4
	1.0	1513.95	17,282.5	40,775.7
	1.5	5595.84	35,765.1	57,023.5
	2.0	5632.69	37,481.8	58,580.1
	2.5	7920.54	38,543.9	68,481.1
r = 5.759 nm	0	274.9	10,996.6	27,214.7
	0.5	814.229	24,012.3	59,720.1
	1.0	6382.94	41,925.1	74,316.3
	1.5	7122.12	43,893	76,289.5
	2.0	9131.92	46,193.1	80,544.8
	2.5	7403.35	50,544.1	77,897.2

**Table 3:** Equivalent grain radius and fraction of grain-boundary atoms in different models.

Model Size/nm	Number of Grains	Total Number of Atoms	Number of Grain-Boundary Atoms	Fraction of Grain-Boundary Atoms/%	Equivalent Grain Radius/nm
20	30	659,793	159,755	24.2	3.993
20	20	663,273	127,411	19.2	4.571
20	10	675,058	102,353	15.3	5.759
30	20	2.26481 × 10^6^	292,352	12.9	6.856
20	5	671,013	82,597	12.3	7.256
30	10	2.27373 × 10 ^6^	243,749	10.7	8.638
40	20	5.3975 × 10 ^6^	537,324	10	9.142
20	2	676,520	59,357	8.8	9.847
30	5	2.28347 × 10 ^6^	185,801	8.1	10.883
40	10	5.39566 × 10 ^6^	427,585	7.9	11.518
40	5	5.43424 × 10 ^6^	338,171	6.2	14.511
30	2	2.29563 × 10 ^6^	129,143	5.6	14.771
40	2	5.4514 × 10 ^6^	245,155	4.5	19.695

**Figure A1:**
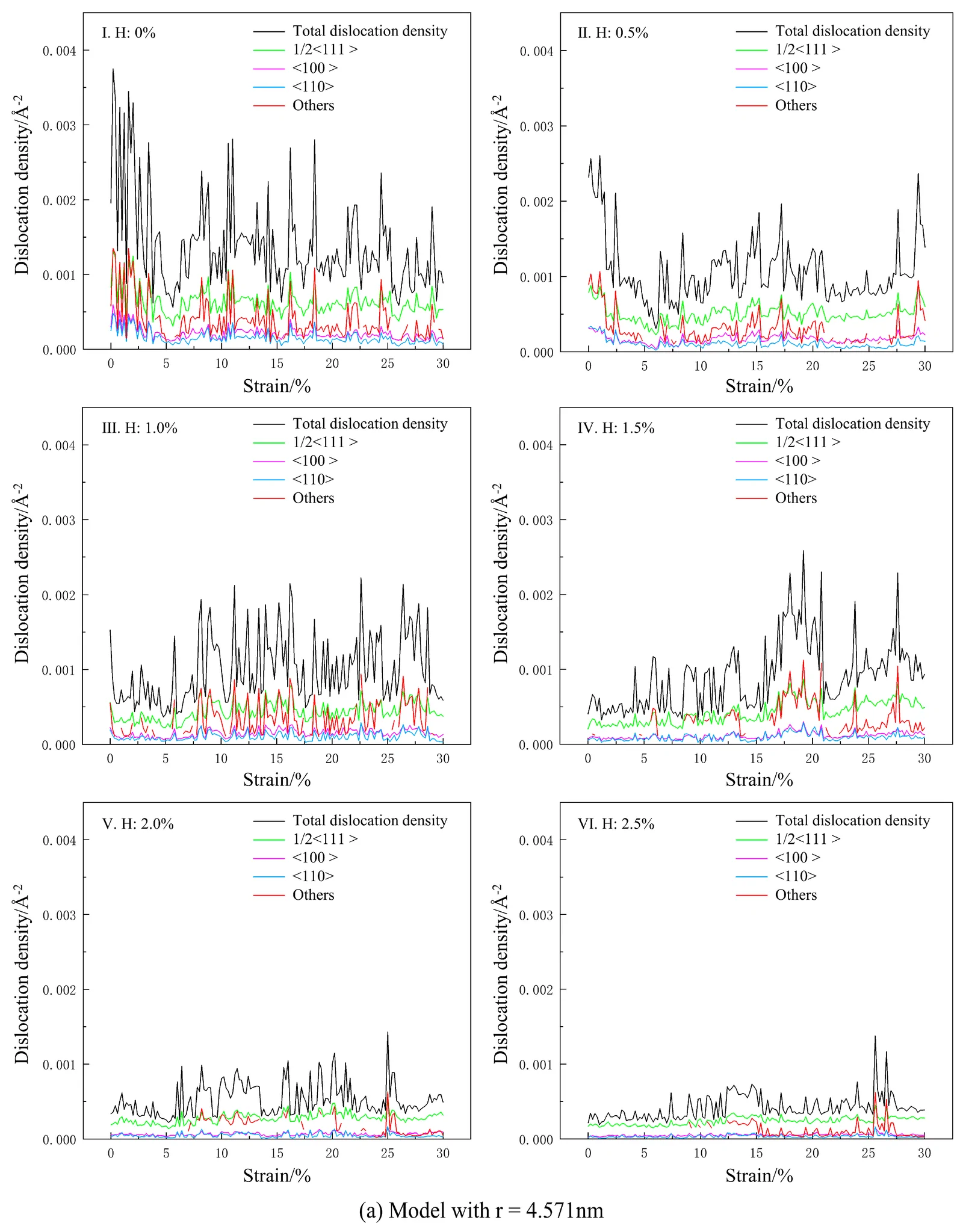

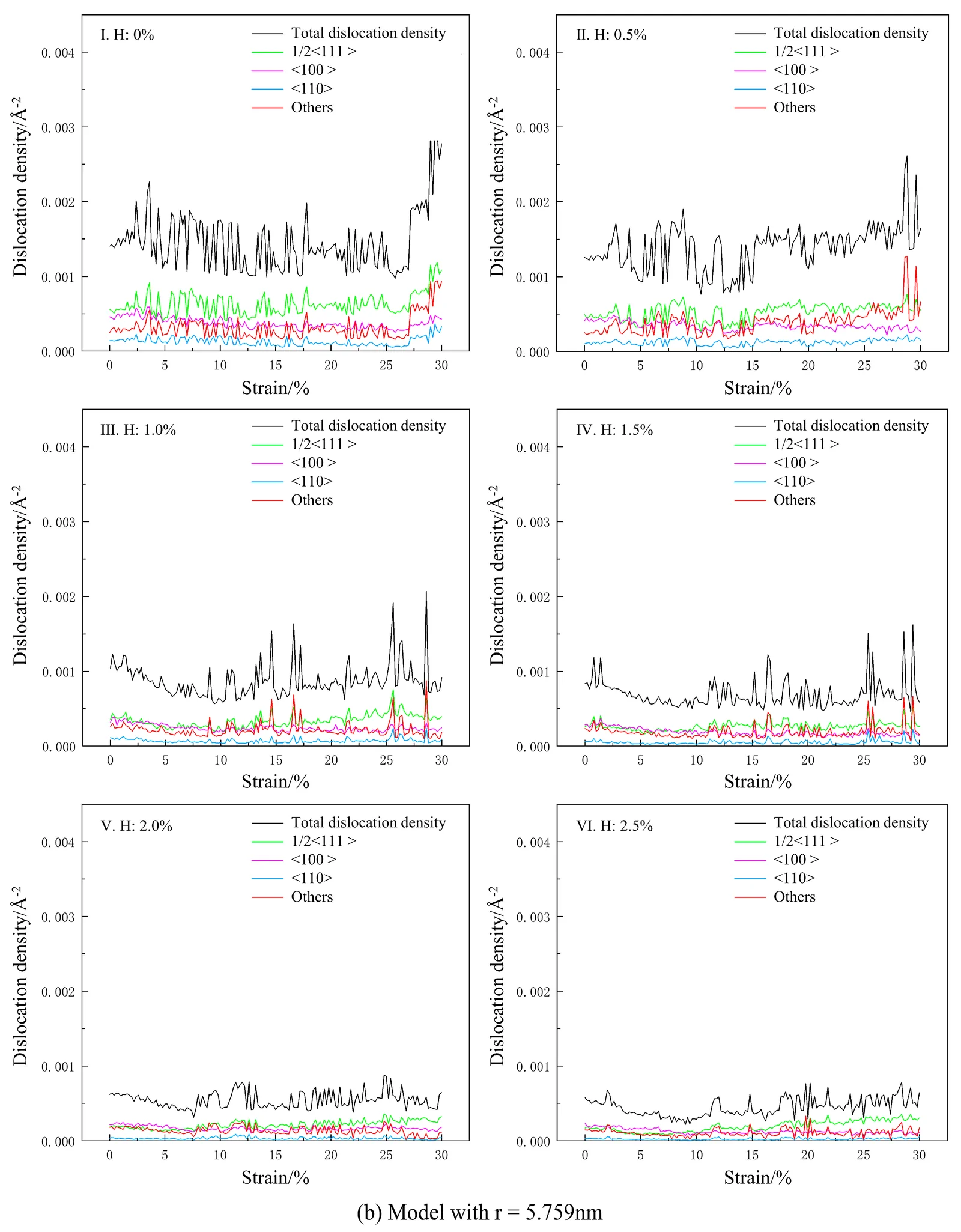
Evolution of different dislocation types during tensile deformation at different hydrogen concentrations: (**a**) model with r = 4.571 nm; (**b**) model with r = 5.759 nm.

## Data Availability

The original contributions presented in this study are included in the article. Further inquiries can be directed to the corresponding authors.

## Abbreviations

The following abbreviations are used in this manuscript:

HELP	Hydrogen-enhanced localized plasticity
HEDE	Hydrogen-enhanced decohesion
HBT	Hydrogen bubble theory
BCC	Body-centered cubic
NPT	Isothermal–isobaric
EAM	Embedded atom method
MSD	Mean squared displacement

## Appendix A. Evolution of Different Dislocation Types During Tensile Deformation

To provide additional information on the dislocation evolution behavior, the detailed changes in different dislocation types for the fine-grained and coarse-grained models are presented here. The dislocations were classified according to their Burgers vectors into 1/2<111>, <100>, <110>, and other types. These detailed results complement the total dislocation density comparison presented in Figure 3 of the main manuscript.
